# Environmental Risk Characterization of an Antiretroviral (ARV) Lamivudine in Ecosystems

**DOI:** 10.3390/ijerph18168358

**Published:** 2021-08-06

**Authors:** Elizabeth Oyinkansola Omotola, Bettina Genthe, Luyanda Ndlela, Olatunde Stephen Olatunji

**Affiliations:** 1 School of Chemistry and Physics, University of KwaZulu-Natal, Durban 4000, South Africa; elizabethoyinomotola@gmail.com; 2 Department of Chemical Sciences, Tai Solarin University of Education, Ijebu Ode PMB 2118, Nigeria; 3 Natural Resources and the Environment Division, Council for Scientific and Industrial Research, Stellenbosch 7599, South Africa; bettinagenthe@gmail.com (B.G.); lndlela@csir.co.za (L.N.)

**Keywords:** bioassays, Ames, Salmonella, *Allium cepa*, *Lactuca sativa*, *Daphnia magna*, emerging contaminants, flora and fauna, toxicity test, mortality rate, chromosomal aberration, mutagenicity

## Abstract

Antiretroviral drugs for the treatment of human immunodeficiency virus (HIV) and other viral infections are among the emerging contaminants considered for ecological risk assessment. These compounds have been reported to be widely distributed in water bodies and other aquatic environments, while data concerning the risk they may pose to unintended non-target species in a different ecosystem (environment) is scanty. In South Africa and other developing countries, lamivudine is one of the common antiretrovirals applied. Despite this, little is known about its environmental impacts as an emerging contaminant. The present study employed a battery of ecotoxicity bioassays to assess the environmental threat lamivudine poses to aquatic fauna and flora. *Daphnia magna* (filter feeders), the Ames bacterial mutagenicity test, *Lactuca sativa* (lettuce) germination test, and the *Allium cepa* root tip assay were conducted, testing lamivudine at two concentrations (10 and 100 µg/L), with environmental relevance. The *Daphnia magna* toxicity test revealed a statistically significant response (*p* << 0.05) with a mortality rate of 85% on exposure to 100 µg/L lamivudine in freshwater, which increased to 100% at 48-h exposure. At lower concentrations of 10 µg/L lamivudine, 90% and 55% survival rates were observed at 24 h and 48 h, respectively. No potential mutagenic effects were observed from the Ames test at both concentrations of lamivudine. *Allium cepa* bioassays revealed a noticeable adverse impact on the root lengths on exposure to 100 µg/L lamivudine. This impact was further investigated through microscopic examination, revealing some chromosomal aberration in the exposed *Allium cepa* root tips. The *Lactuca sativa* bioassay showed a slight adverse impact on both the germination rate of the seeds and their respective hypocotyl lengths compared to the control. Overall, this indicates that lamivudine poses an ecological health risk at different trophic levels, to both flora and fauna, at concentrations previously found in the environment.

## 1. Introduction

It has been reported that pharmaceuticals are in continuous release into the environment from different point sources, thereby contaminating water bodies [1,2]. Once these pollutants enter the environment, they can affect living organisms at many trophic levels in the ecosystem [3]. Bioindicators serve as a critical integral part of assessing these environmental impacts. The adverse effects of a number of active pharmaceutical residues investigated on various organisms include deoxyribonucleic acid (DNA) fragmentation, modulation of lipid metabolisms, alteration of immunological parameters such as lysosomal membrane stability [4]; changes in gene expression, apoptosis, delay in hatching, abnormal spinal cord formation, heart and yolk sac abnormalities, pericardial edema, anomalous eye and tail formation, and decrease of the heartbeat in *Danio rerio* [5]; endocrine disruption and genetic alteration in *Mytilus galloprovincialis* [6]; inhibition of luminescence of bacteria in *Vibrio fischeri* [7] and contraction and disintegration of *Hydra magnipapillata* [8].

One of the pharmaceutical compounds detected in aqueous environments in a recent screening survey in South Africa is lamivudine (LMV) [9]. LMV is a nucleoside reverse transcriptase inhibitor (NRTI) used in antiretroviral therapy (ART) for the treatment of human immunodeficiency virus (HIV) infections [10]. Its mechanism of action includes blocking reverse transcriptase enzyme activity, thus, causing pro-viral nucleic acid chain termination [10]. Lamivudine is also an orally active anti-hepatitis B Virus (anti-HBV) drug [11], which acts via the suppression of HBV replication, thus reducing liver disease progression [11]. As of the year 2015, lamivudine remained the only dual-purpose drug used to treat both HIV and HBV in Uganda [12], mainly because HIV patients are usually susceptible to other infections, including HBV. This can be attributed to the fact that both viruses share modes of transmission [12], thus, increasing the chances of co-infection.

The global administration of lamivudine in HIV therapy is widespread, especially in countries such as the United States of America and South Africa, where the prevalence of HIV infection is high. The current population of people living with HIV, in South Africa is estimated to be over 7 million, with an annual estimate of 240,000 new infections (https://www.avert.org/professionals/hiv-around-world/sub-saharan-africa/south-africa, accessed on 10 June 2021). This implies high lamivudine and other ARV drug usage, and hence the potential for post-consumption release/discharge from point sources such as wastewater treatment plants, among others, into the environment. Their presence in the environment, such as water bodies, is further compounded by inefficient sewage treatment plants, with additional treatment methods required to remove it in conventional wastewater treatment [13]. In countries with currently inefficient wastewater treatment facilities, there is a high likelihood of environmental release and limited infrastructure, presenting multiple challenges. In a report presented by Osunmakinde et al. [14], lamivudine was listed as one of the top 20 most prescribed drugs in South Africa. Wood, Swanepoel, and Abafe et al. [1,15,16] reported the occurrence of lamivudine at 0.89, 242, and 130 ng/L in some African environments, respectively, while K’oreje and Ngumba [17,18] indicated significantly higher concentrations of 167,000 and 5428 ng/L, respectively, in Kenya. Nannou et al. [19] noted that there are only 13 reports on the presence of antiviral drugs in Africa’s aquatic environment, albeit no data is available on the ecotoxicity of lamivudine. Despite the numerous reports of lamivudine human and animal pharmacokinetics and toxicology, information concerning its environmental kinetics and toxicology is lacking. There is a need to investigate the occurrence levels of lamivudine and characterize its fate and environmental behavior in order to be able to estimate the potential environmental risk they pose to exposed non-target native organisms in different matrices. This study is the first report on the ecotoxicity assessment of the antiretroviral drug-lamivudine, employing this combined battery of bioassays to the best of our knowledge.

This study assessed the risk that active pharmaceutical residue-lamivudine might pose to living organisms when released into the environment. In order to achieve this, a battery of assays was employed, including the following: *Daphnia magna* 24–48-h toxicity test, the Ames Salmonella mutagenicity test, the *Lactuca sativa* seed germination test, and the *Allium cepa* root assay. Each bioassay was selected because of its association with a different biological component, making them a good representation of different trophic levels, with relevance to agriculture and freshwater. These bioassays were conducted making use of a control and lamivudine at two concentrations (10 and 100 µg/L), with environmental relevance. These concentrations were chosen as they represent the range of concentrations reported in the authors’ previous work [9]. In the previous work by Omotola and Olatunji [9], these concentrations were analytically confirmed on the liquid chromatography mass spectrometer (LCMS) during environmental monitoring of lamivudine in the KwaZulu-Natal Province of South Africa.

## 2. Materials and Methods

### 2.1. Materials

Lamivudine standard (DLD Scientific: Cas No. 134678-17—USP34 grade) was purchased from J and H Chemical Co Ltd., Durban, South Africa. Analytical grade chemicals and solvents, including agar-agar, D(+) glucose monohydrate, and NaCl (for the preparation of minimal and top agar), MgSO_4_.7H_2_O, citric acid.H_2_O, K_2_HPO_4_, NaNH_4_HPO_4_.4H_2_O, L-histidine, D-biotin, and methanol, were procured from Sigma Aldrich, Stellenbosch, South Africa. Daphtoxkit, with kit number DM576, ephippia (batch number-DM07119), and spirulina (batch number SP300118) were purchased from Tox Solutions Kits and Services, Stellenbosch, South Africa), while *Lactuca sativa* seeds were purchased from South African nurseries. The EPBI Muta-ChromoPlate^TM^ was also procured from, Stellenbosch, South Africa. Cool white light bulb for the illumination of ephippia was bought from South Africa. MilliQ water used in the LC analysis was prepared using RiOs^TM^/Elix 5 Millipore Water Purifier (Model No PF05113). Whatman filter paper, Petri-dishes, and aquarium air pump were provided by the Council for Scientific and Industrial Research (CSIR), Stellenbosch, South Africa. All experiments were carried out at CSIR laboratory, Stellenbosch, South Africa, while microscopic examination was carried out at the Genetics Department, Stellenbosch University, South Africa.

### 2.2. Methods

#### 2.2.1. Data Interpretation

Descriptive statistics such as sum, mean, standard deviation, and parametric statistics such as analysis of variance (ANOVA) were conducted by using Microsoft Excel 2016 package. Prior to these tests, the skewness, kurtosis, and normality of the data were tested using statistical package for social sciences (SPSS). The data did not differ from the Gaussian distribution model.

#### 2.2.2. *Daphnia magna* Acute Toxicity Test

Acute immobilization test with *Daphnia magna* (freshwater fleas) was carried out according to the Organization for Economic Cooperation and Development (OECD) guidelines for the testing of chemicals, Tests No. 202 [20], which forms the basis of the kits manufacturer’s protocol, with slight modification. The basic principle of this test is based on whether the test solutions are toxic to the daphnids; signified by motility and mortality rates.

Freshwater was simulated using standard concentrated salt solutions (batch number ISOD070319). Sodium hydrogen carbonate (NaHCO_3_), calcium chloride dihydrate (CaCl_2_.2H_2_O), magnesium sulfate heptahydrate (MgSO_4_.7H_2_O), and potassium chloride (KCl) concentrated solutions were used to simulate the freshwater medium for the daphnids’ hatching and exposure study. Ephippia were hatched within 72 h period at 28 °C under constant illumination with the aid of cool white light. After the 72-h hatching period, the daphnids were fed with dry algae (spirulina) for a period of 2 h (this was to annul external effects, such as starvation, prior to exposure to lamivudine), after which they were harvested into a 30-well plate containing the test solutions. Test solutions of lamivudine (10 and 100 µg/L) were prepared in freshwater using serial dilutions from a 1000 µg/mL stock solution of lamivudine. Freshwater (containing the same amount of methanol used in the preparation of the test solutions) was used as the control. Freshwater used for both control and exposure studies was aerated with the aid of an aquarium air pump before exposure. Four replicate analyses were conducted for each test solution where five active daphnids were placed in each well (making a total of 20 daphnids) and exposed over a period of 24 and 48 h, with survival rates from which the lethal dose (LD50) was obtained.

#### 2.2.3. Ames Mutagenicity Test

Bacterial reverse mutation test detects mutations that reverse mutate test strains (*Salmonella typhimurium*) and restore the functional capability of the bacteria to synthesize an essential amino acid required by the parent test strain [21]. The revertant bacteria are detected by their ability to grow in the absence of the amino acid required by the parent test strain. Two methods were employed to conduct the Ames mutagenicity test using 3 different *S. typhimurium* bacteria strains (TA98, TA100, and TA100—P450, which simulates the metabolic activation of rat liver fraction S9), to account for discrepancies in the way similar organisms (bacteria) react to lamivudine in the environment.

Overnight cultures of bacteria were prepared in a fresh nutrient broth medium (Merck, Darmstadt, Germany), and their growth was confirmed by turbidity. The tests were performed using the original agar-plate test method described by Ames et al. [21] and included in standard methods for the examination of water and wastewater [22].

A second test for comparison was run using the EPBI Muta-ChromoPlateTM. The test makes use of a 96-well microplate version of the *S. typhimurium* Ames Test [21] developed to test mutagenic materials in water, foods, and chemicals. It is a quick but expensive test kit for this assay. Only one bacterial strain type (TA 100) was used for comparison with the agar plate method. A minimal medium containing histidine and biotin that allow for a few cell divisions was used. Bacteria (100 µL) grown overnight at 37 °C were used for the study. Positive (sodium azide) and negative controls were included, and the measurement of the background (bacterial strain + reaction mixture only) reverse mutation rate was compared to the test sample mutation rates after exposure. Generally, if samples have twice the number of reverse mutations compared to the background (or spontaneous) mutation rate, the test sample is considered mutagenic [21].

#### 2.2.4. *Lactuca Sativa* Bioassay

The *Lactuca sativa* (lettuce) seed bioassay was carried out according to the method employed by Steyn et al. [23], which relies on lettuce seed bioassay of the US Environmental Protection Agency E.P.A. [24], with slight modifications. Briefly, the test was conducted in 90 by 15 mm Petri dishes (Lasec, Stellenbosch, South Africa) without sediment or soil. Twenty lettuce seeds (Starke Ayres, Stellenbosch, South Africa) of similar shapes and sizes were cultivated on filter paper. Prior to this, the filter paper was moistened with 3–5 mL of lamivudine test solutions of different concentrations (10 and 100 µg/L). The control solvent was water. Afterward, the Petri dishes were optic-blinded with aluminum foil and kept in the dark at ambient temperature for the 120 h duration of the experiment. This was performed to avoid bias from the impact of external factors such as sunlight on the germination/growth rate of the seeds. After the test duration elapsed, the number of germinated seeds and their respective hypocotyl lengths were counted, measured, and compared to the control. Germination rates were calculated using the equation GR = GSS/GSC, as reported by Priac et al. [25]; where GSS and GSC are the numbers of germinated seeds in the sample and control, respectively.

#### 2.2.5. *Allium cepa* Root Tip Assay (Onion Root)

The onion root tip assay was carried out according to the method described by Barbério [26] with slight modifications. Whole onions test specimens were grown at room temperature (in triplicate). Onions were initially grown for two days in tap water. Prior to this, the ring of the root, primordial at the bottom of the onion bulb, was scraped with a sterile surgical blade to remove all existing root growth. After the two days of tap water exposure (tap water was used as the control), the lengths of each root were recorded, followed by exposure to test solutions for another two days. Under controlled conditions, it is expected that the root length should double. Hence, both values (the most extended root length before and after exposure to lamivudine solution) were used as a criterion for ascertaining any adverse effects on the onion roots. At the end of the exposure, the onion roots were cut at 3 cm from the tip and placed in Carnoy’s fixative (3 parts glacial acetic acid to 1-part absolute ethanol (Merck, Darmstadt, Germany)) until further microscopic analysis.

For microscopic examination, the fixed roots were washed in distilled water and then hydrolyzed by placing in 1 M hydrochloric acid (HCl) in a water bath at 60 °C for 7.5 min to dissolve the substances that unite the onion root cell walls, usually pectin [27]. Root tips were washed in distilled water, after which they were stained using Feulgen stain for 2–3 min. Root tips were then rinsed in distilled water and transferred to clean glass microscope slides, and counter-stained with acetocarmine. The stained root tips were first crushed with the flat end of the surgical blade [27] and then under a coverslip, applying pressure with care to avoid movement of the coverslip or breaking the coverslip. The squashed onion root tips were thereafter imaged using a LEICA DM-750 light microscope at 400× and 1000× magnification. Qualitative analysis was carried out by observing the abnormalities of the onion chromosomes as reported by Çavuşoğlu et al. [28] and comparing them to control chromosomes.

## 3. Results and Discussion

Assessing the potential environmental impacts of widely used emerging contaminants requires sensitive, robust, and feasible testing which is reproducible. The bioindicators selected in this study fit these criteria, with relevance to agricultural and freshwater impacts of lamivudine in the environment.

### 3.1. Daphnia Acute Toxicity Test

The *Daphnia magna* are reported to be very sensitive freshwater crustaceans that are quick to respond to small changes in water chemistry; hence they are used as a target indicator organism in exposure studies. Previously, these filter feeders have indicated acute and chronic exposure responses to environmental toxins, acid mine drainage, and flocculants [29]. Results showed that 10 µg/L lamivudine had higher neonates’ survivals relative to the control (same amount of methanol in test solution as in aerated freshwater), while neonates exposed to 100 µg/L lamivudine test solution had a lower survival rate (S1).

There were substantial significant differences between the neonates’ survival rates in the test solutions relative to the control (*p* << 0.05, *p* = 3.29 × 10^−10^). A 10% mortality was observed in 10 µg/L lamivudine test solution at 24 h, and the mortality rate increased to 45% at 48 h. An 85% mortality was noted in 100 µg/L lamivudine test solution at 24 h, and 100% at 48 h. Neonates in the control test solution had 100% survival after 24 and 48-h exposure; thus, there was no toxicity response. Lower concentrations of lamivudine (10 µg/L) were more toxic with exposure over time, with a 35% increase in mortality over 48 h, whilst higher concentrations had more immediate acute impacts (100 µg/L). Earlier studies assessing the behavioral response of *Daphnia magna* to nano-metal oxides and chemicals found that daphnids were actively ingesting nanoparticles at feeding and fasting intervals and that chemical concentrations elicited a greater response than the actual structure/toxicity, respectively [30]. The findings in this study corroborate this, with higher concentrations having impacts that are more acute. Moreover, having been fed prior to exposure-to eliminate starvation as a mortality cause, active ingestion is possibly the route of exposure, corroborating the findings of Ren et al. [31]. The increased mortality overtime at 10 µg/L is an indication that the presence of lamivudine in the environment can potentially cause both acute and chronic adverse effects on aquatic organisms.

**Figure 1:**
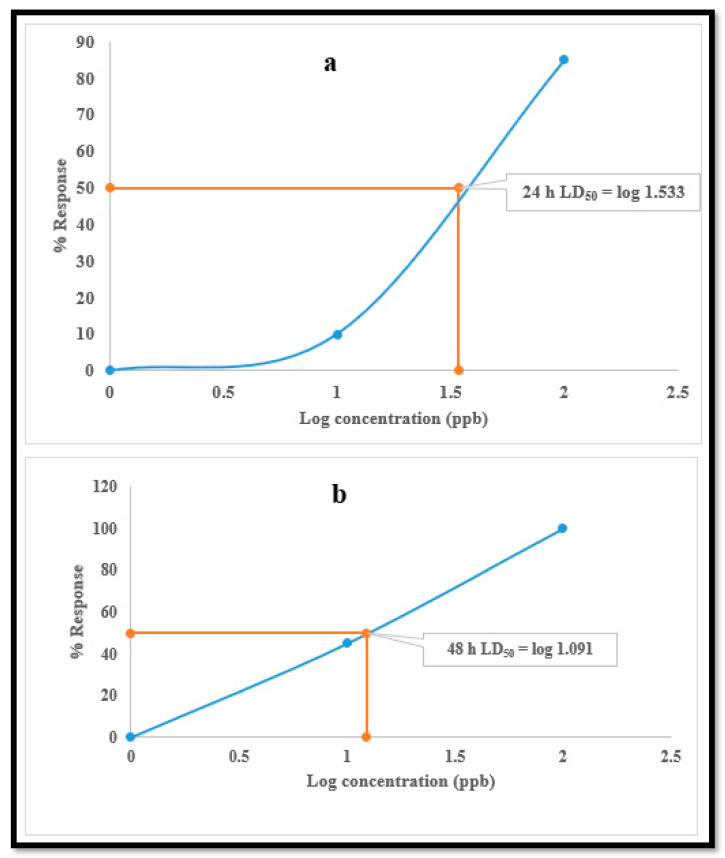
LD50 of lamivudine at; (**a**) 24 h and (**b**) 48 h.

The LD50 calculations for the test samples indicated that exposure to the one-third equivalent of LD50 concentration (34.1 ug/L) at 24 h had a more detrimental impact (LD50) on the daphnids at 48 h (12.3 µg/L) (Figure 1), with a typically monotonic response to lamivudine.

### 3.2. Ames Mutagenicity Test

Mutagenic substances, referred to as genotoxins, can cause changes to the DNA structure. Such changes can result in distorting the transcription and replication of the DNA, thus leading to cell death [32]. The Ames Salmonella test is an essential bioassay used to test for the mutagenicity of toxic materials through the exposure of bacteria for a period of 3 to 5 days. Where a substance or compound shows potentials for altering the DNA structure of living organisms in such a way that it inhibits normal functions of organs, such substance is classified as a mutagen. A phenomenon such as this could eventually lead to the death of living entities exposed to the mutagens. Lamivudine was tested using this assay to determine its mutagenic effects.

**Figure 2:**
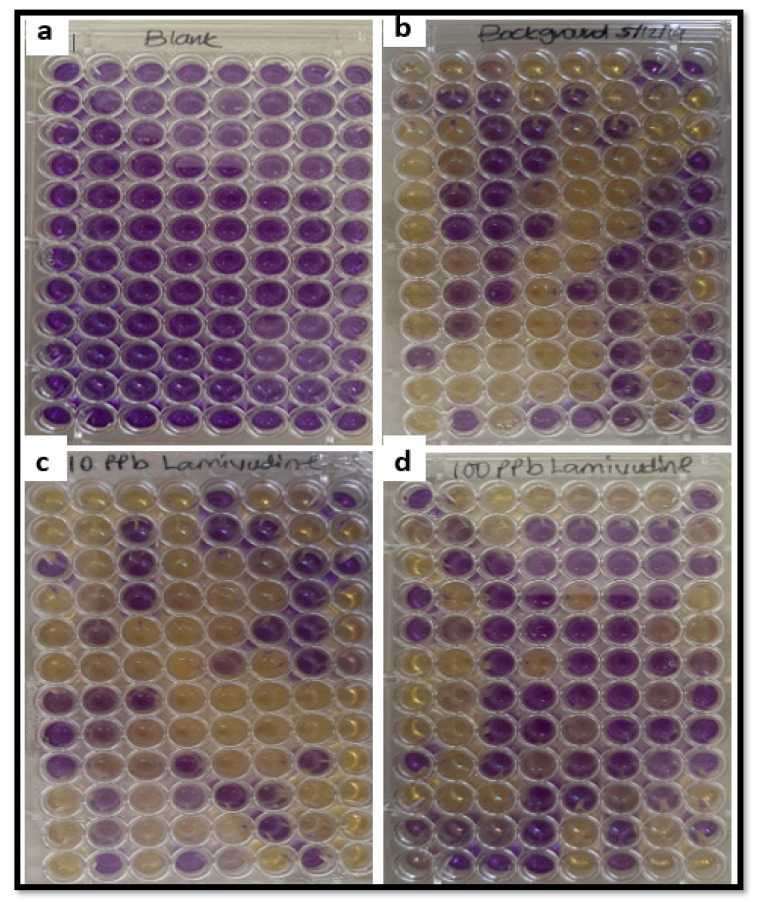
Ames mutagenicity test (EPBI^TM^) using TA 100 *S. typhimurium* bacterial strain: exposure to test solutions; (**a**) blank, (**b**) background, (**c**) 10 µg/L lamivudine, and (**d**) 100 µg/L lamivudine.

Results from TA 100 strain of *S. typhimurium* bacterial exposure to lamivudine using the EPBI Kit^TM^ (Stellenbosch, South Africa) indicated that both 10 and 100 µg/L lamivudine test solutions are not mutagenic when compared to the background (Figure 2). Experimental observations indicated no mutation response as measured by the number of wells that turned purple to yellow of 96-wells in the EPBI Kit^TM^ plate after six days of exposure. The conversion of well from purple to yellow is a function of the mutagenic activity rate on *S. typhimurium* TA 100 exposed to lamivudine. All wells in the positive control plate indicated a positive response, with all wells turning yellow on exposure of *S. typhimurium* TA 100 to sodium azide, a known mutagen. Negative responses to the background, 10 and 100 µg/L lamivudine exposure in wells of the EPBI KitTM plates (Figure 2a–d) were counted and compared.

**Figure 3:**
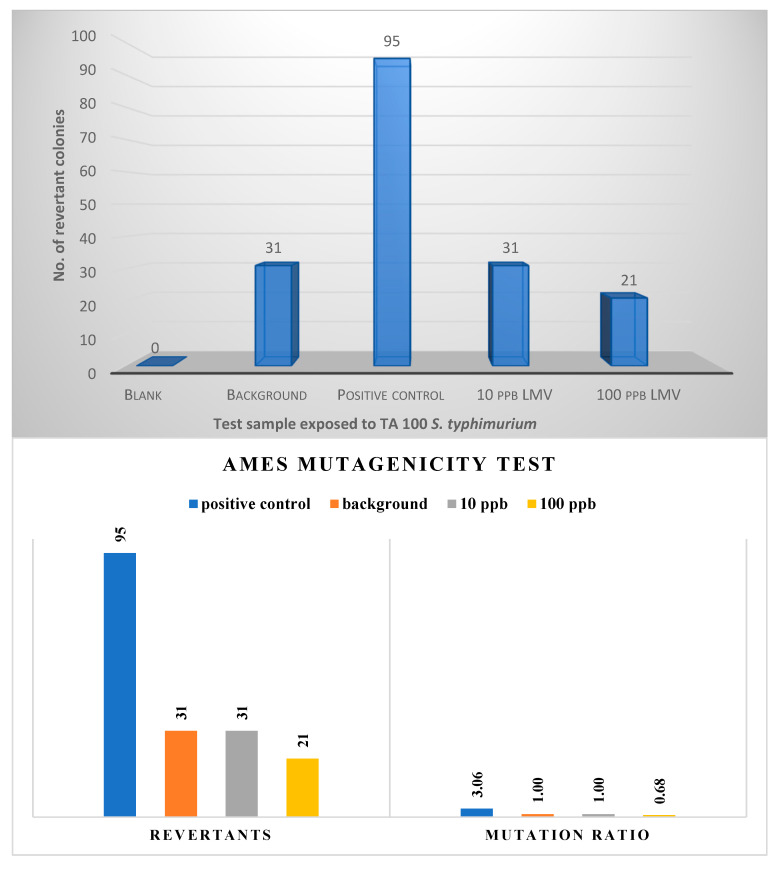
Result of mutation rate obtained from Ames test using EPBI kit.

A substance is deemed mutagenic when the mutation rate of the substance is twice that of the background. Study results (Figure 3) revealed that the mutation rate of *S. typhimurium* TA 100 bacteria strain exposed to lamivudine test solution at 10 µg/L and 100 µg/L levels is not twice that of the background; hence lamivudine is not considered a mutagen.

In addition to the test kit method, the agar plate method used in toxicity testing until recently was also used as a complementary method [21,33]. This method is inexpensive compared to the kit and allows for multiple testing of toxic materials. However, it is labor-intensive and rigorous relative to the kit. Results from the agar plate test (S2) as well confirmed lamivudine to be non-mutagenic to *S. typhimurium* TA 100 strain. Results (S3) revealed that the mutation rate of *S. typhimurium* TA 100 bacteria strain exposed to lamivudine test solutions is not twice that of the background and hence not considered a mutagen. This affirms the result obtained from using the EPBI kit.

**Figure 4:**
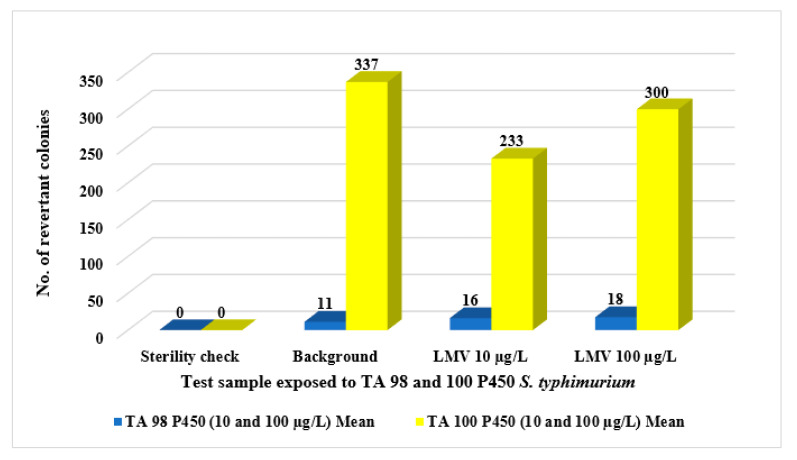
Ames Salmonella mutagenicity test: comparison between responses of TA 98 P450 and TA 100 P450 bacterial strains to lamivudine test samples.

Further exposure studies using two other *S. typhimurium* bacterial strains, TA98 P450 and TA100 P450, were carried out (using the agar plate method) to ascertain lamivudine’s non-mutagenicity to different bacterial test strains with simulated metabolic activation of rat liver fraction, S9. Results from this exposure study revealed a higher number of background bacteria colonies as compared to those of test samples (Figure 4). Thus, *S. typhimurium* TA 98 P450 and TA100 P450 bacterial strains were not susceptible to lamivudine at the exposure concentrations 10 and 100 µg/L. Based on this, lamivudine indicated no mutagenic impacts, which would otherwise be linked to possible carcinogenic effects.

### 3.3. Lactuca Sativa (Lettuce Seeds) Bioassay

A simple, quick, cost-effective, and sensitive *Lactuca sativa* seed bioassay was used to evaluate the potential environmental risk of lamivudine [25,34]. *L. sativa* seeds are one of the plants’ specimens recommended by the United States Food and Drug Administration [35] to assess possible risks contaminant could pose to living organisms. The test is based on an analysis of the phytotoxic effects of contaminants in the germination phase of the seeds and the development of the seedlings during a 5-day period of exposure.

Findings from the germination of lettuce seeds bioassay indicated that there were slight or minimal percentage variations of less than 6% difference in seed germination between the control and 10 µg/L lamivudine test samples after exposure. The difference between percent germination of lettuce seed exposed to 100 µg/L lamivudine test solution in comparison with the control test solution was 10% (S4).

Furthermore, one-way ANOVA showed that there was no statistically significant difference (*p* > 0.05, *p* = 0.10) between lengths of lettuce seed plantlets (hypocotyl) exposed to 10 µg/L lamivudine and that of the control test solution. ANOVA conducted on the results obtained from lettuce seed germinated on treatment with 100 µg/L lamivudine solution showed considerable difference (*p* < 0.05, *p* = 0.01) as compared to that of 10 µg/L lamivudine solution and that of the control solution. As recommended by the normalized method, the germination rate under 90% for the control conditions is unacceptable [25]. Hence, the values obtained are accurate, precise, and reliable. The sensitivity of *Lactuca sativa* has been determined against heavy chemicals, among others [36], with a recommendation of 80% germination or less as a signal of sensitivity [37], which was not observed in this study. *Lactuca sativa* has been recommended as an ideal bio-indicator for some compounds; however, testing of certain compounds does not directly inhibit the germination or the root length in this instance. Additionally, the relevance to agriculture and irrigation in lettuce has been noted in earlier studies which also observed the safety of water-based on coliform presence in irrigation water [38]. Prior recommendations by Priac et al. [25] mention the varying sensitivity of cultivars, whilst Ndlela et al. [29] found reduced sensitivity to environmental toxins at low concentrations compared to other bioindicators. This highlights the importance of bioindicator suitability and the value of testing more than one bioindicator when assessing the environmental impacts of emerging contaminants.

### 3.4. Allium cepa Root Assay

The *Allium cepa* (*A. cepa*) root is a suitable bioindicator for the assessment of the potential genotoxic agents that may be present in the environment [27,39]. Their phytotoxic suitability has been attributed to their relatively large size and appropriateness for the detection of morphological changes [40]. The onion root assay was achieved through the observation of root lengths (mm) on exposure to 10 and 100 µg/L lamivudine test samples, while tap water was used as a control solution (Table 1).

One-way ANOVA revealed no significant difference (*p* > 0.05, *p* = 0.18) between the changes in the length of the 10 µg/L lamivudine treated *A. cepa* onion root tips and that of the control solution treated *A. cepa* roots, while a noticeable significant difference was observed with 100 µg/L (*p* < 0.05, *p* = 3.60 × 10^−3^) (Table 1). A reduction of approximately 22% and 43% was observed in the 10 µg/L and 100 µg/L, respectively.

**Figure 5:**
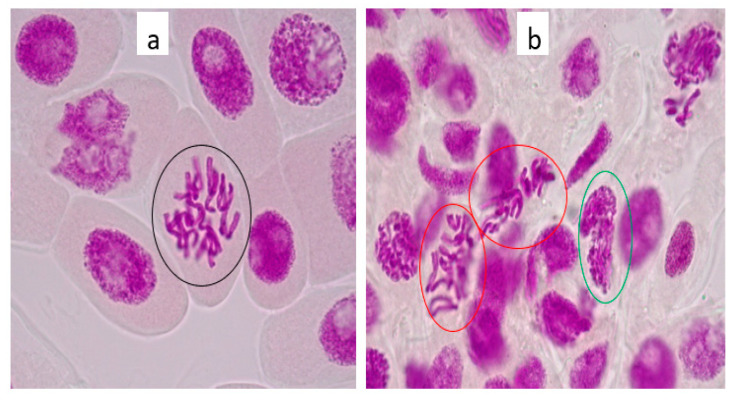
(**a**) tap water control cells showed normal mitosis phase (anaphase) in root tip meristem indicated by the black encircled picture. (**b**) showed irregular prophase and metaphase in root tip meristem cells of *Allium cepa* exposed to 100 µg/L lamivudine, indicated by the red and green encircled pictures, respectively. (Onion root tip imaged at 1000× magnification).

In order to ascertain the genotoxic potential of 100 µg/L lamivudine on the root lengths of the onion, chromosomes taken from the meristematic cells of onion roots were studied via microscopic examination (Figure 5a,b).

Figure 5a showed untreated onion root tips exposed to tap water (control) compared to onion root tips treated with 100 µg/L lamivudine test solution in Figure 5b. These were taken as representative samples to indicate the differences in the mitotic stages of the onion roots exposed to the test solution. This result revealed an aberration in the mitotic stage of the onion cell when compared to the control. This aberration was referred to as ‘irregular anaphase’ by Çavuşoğlu et al. [28]. The effects of lamivudine on *Allium cepa* were observed at macroscopic and chromosomal levels. Although the observed effects were not quantified for the determination of mitotic indexing, aberrations were clearly observed in numerous nuclei exposed to lamivudine. Being among the staple vegetables of economic value and a recommended bio-indicator [41], the genotoxic impacts of lamivudine on onion cells are concerning from an agricultural yield and human consumption perspective.

### 3.5. Lamivudine Exposure Characterization and Evaluation

In order to assess the exposure risk lamivudine pose to the environment, four organisms were tested in the exposure study. These organisms include *Daphnia magna*, *Salmonella typhimurium*, *Lactuca sativa*, and *Allium cepa*. Results from exposure studies and risk evaluation of lamivudine on the four organisms are summarized (Table 2).

*Daphnia magna* toxicity test revealed a mortality rate of 85% on exposure to 100 µg/L lamivudine in freshwater, which increased to 100% at 48-h exposure, whereas the control daphnids in simulated freshwater were 100% mobile and active. However, *D. magna* exposed to a lower concentration of 10 µg/L lamivudine for 24 h and 48 h periods had 90% and 55% survival rates, respectively. The Ames Salmonella mutagenicity test showed no potential mutagenic effect. *A. cepa* bioassay revealed a noticeable adverse impact on the root lengths on exposure to 100 µg/L lamivudine. This impact was further investigated through microscopic examination, which revealed some chromosomal aberration in the *A. cepa* root tips. A 120-h seed germination test using *Lactuca sativa* seeds in test solutions of the same concentration levels showed a slight negative impact on both the germination rate of the seeds and their respective hypocotyl lengths compared to the control.

Thus, there was an injury-related response in *D. magna* survival, germinating rate of *L. sativa* seeds, and *A. cepa* root tip mitotic stage. This implies that lamivudine may pose an ecological health risk to both flora and fauna at concentrations previously found in the environment. It is unclear as to whether the injury-induced and/or the environmental threat posed by lamivudine can be classified as mild or severe (substantial). It is therefore imperative that the pathway analysis, analysis of critical components in the injury phase and ecological risk assessment of lamivudine forms part of natural resource damage assessment. While the habitat equivalency analysis did not suggest the need for damage assessment due to lack of evidence of impairment of functions, chemical and toxicity-based approach is needed for assessment of interrelated functions and qualitative estimate of hazard and risk associated with residues of lamivudine in various matrices.

## 4. Conclusions

The present study revealed that lamivudine had a quantifiable adverse impact on *Daphnia magna* and *Allium cepa*. This was confirmed by a hundred percent mortality rate of the daphnids after the 48-h exposure to 100 µg/L lamivudine and the root lengths as well as chromosomal anomalies in the onion. Although lamivudine’s mechanism of action involves the inhibition of viral DNA synthesis via reverse transcriptase DNA chain termination, its’ mechanism of action has not been investigated on *Daphnia magna* and *Allium cepa*. However, the toxic effect of lamivudine on the organisms tested in both assays may probably result from a failure of cell replication. Ames test showed lamivudine is not a potential mutagen, on the exposure of three different bacterial strains, with and without S9, to 10 and 100 µg/L lamivudine solution, since the mutation rates of the test samples were not twice that of the background. However, potential chronic mutagenic effect of lamivudine should not be ruled out. As per *Lactuca sativa* bioassay, it was revealed that the germination rate differed between 100 µg/L lamivudine test solutions compared to the control. Considering the volumes of lamivudine ingested and subsequently released into the environment, this study highlights both the environmental impacts and the potential seriousness of lamivudine as an emerging contaminant. This is particularly of concern in countries that have not adopted the additional phosphate removal step in water treatment, which has been found effective in reducing lamivudine by over 60%. The re-circulation and, therefore, compounding of this antiretroviral without removal map acute, chronic, and repeated secondary exposure of all water users from aquatic life, agriculture, and ultimately human ingestion. A review by Ncube et al. [42] highlighted numerous major concerns, some of which are addressed, stressed, and corroborated in this research. Firstly, the limitation in ecotoxicity testing of antiretrovirals, although some of the greatest antiretroviral programs are in African countries. Secondly, the potential health impacts that have clustered antiretrovirals as more dangerous than other common hazardous compounds and lastly, the possible drug-drug interactions that can occur from exposure to antiretrovirals in the environment [42]. This research provided a combination of bioassays in testing one of the common emerging contaminants, for which there is limited data. Furthermore, the bioassays conducted indicated the variation in indicator sensitivity and, therefore, suitability in testing certain compounds. Lastly, sensitivity was observed in bioindicators from freshwater and agricultural relevance, therefore emphasizing the value of further research in the understanding and mitigation of these emerging contaminants. Thus, lamivudine can be considered to pose a risk to both aquatic flora and fauna; however, these risks may differ with respect to living entities.

## Figures and Tables

**Table 1:** *Allium cepa* root length before and after exposure to lamivudine solutions.

Test Solution	RLAE/RLBE			Mean RLAE/RLBE	*p* Values Relative to Control
	Sample 1	Sample 2	Sample 3		
Control	2.00	2.37	2.31	2.23 ± 0.20	
10 µg/L LMV	1.69	2.25	1.30	1.75 ± 0.48	0.1818
100 µg/L LMV	1.50	1.20	125	1.32 ± 0.16	3.60 × 10^−3^

RLAE—most extended root length after exposure; RLBE—most extended root length before exposure.

**Table 2:** Summary of exposure risk evaluation of lamivudine.

Test Solution	*Daphnia magna* Bioassay			Ames Mutagenicity Test (NBCS/NBCG)			*Lactuca Sativa* Bioassay			*Allium cepa* Bioassay		
	24-h Mean % Mortality	48-h Mean % Mortality	*p* Values	TA 98 P450	TA 100	TA 100 P450	% Germination	Mean Length (mm)	*p* Values	Mean RLAE/RLBE	*p* Values	Microscopic Analysis
Control	0	0		0/11	0/153	0/337	93.35	54.00 ± 12.78		2.23 ± 0.20		Regular anaphase
10 µg/L	10	45	2.76 × 10^−6^	16/11	160/153	233/337	88.35	58.67 ± 8.08	0.10	1.75 ± 0.48	0.1818	
100 µg/L	85	100	1.11 × 10^−3^	18/11	163/153	300/337	83.35	49.67 ± 6.51	0.01	1.32 ± 0.16	3.60 × 10^−3^	Irregular anaphase

Codes: NBCS/NBCG—number of *S. typhimurium* bacteria colonies exposed to test sample per number of bacteria colonies from background result; *p* values at 95% confidence level; Grey areas indicate no value/result.
